# Supplementary material for: From sequence to enzyme mechanism using multi-label machine learning
Source: BMC Bioinformatics. 2014 May 19;15:150. doi: 10.1186/1471-2105-15-150 (PMC4229970; doi:10.1186/1471-2105-15-150)
Supplement: Additional file 2 — Java code of ml2db. Additional file ml2db_code.tar.gz contains the Java source code to run the multi-label machine learning experiments and save the results to database. The code’s Javadoc is included. [file 1471-2105-15-150-S2.zip › additional file 2/ml2db/ml2db/doc/main/ml2db/mulan/mulanarff/class-use/IdMultiLabelInstances.html]

Uses of Class main.ml2db.mulan.mulanarff.IdMultiLabelInstances


JavaScript is disabled on your browser.


- Overview
- Package
- Class
- Use
- Tree
- Deprecated
- Index
- Help

- Prev
- Next

- Frames
- No Frames

- All Classes

## Uses of Class main.ml2db.mulan.mulanarff.IdMultiLabelInstances

- Packages that use IdMultiLabelInstances

  | Package | Description |
  | --- | --- |
  |  |  |
  | --- | --- |
  | main.ml2db.al |  |
  | main.ml2db.al.learning |  |
  | main.ml2db.al.utility |  |
  | main.ml2db.al.utility.confidence |  |
  | main.ml2db.al.utility.globaldistance |  |
  | main.ml2db.al.utility.hybrid |  |
  | main.ml2db.al.utility.neighbours |  |
  | main.ml2db.al.utility.pickers |  |
  | main.ml2db.al.utility.pickers.byfrequency |  |
  | main.ml2db.graphviz |  |
  | main.ml2db.mulan.learn.crosseval |  |
  | main.ml2db.mulan.learn.traintest |  |
  | main.ml2db.mulan.mulanarff |  |
  | main.ml2db.mulan.mulanarff.database |  |
  | main.ml2db.mulan.predict |  |
  | main.ml2db.weka |  |
  | test.ml2db.al.utility.confidence |  |
  | test.ml2db.mulan.mulanarff |  |
- - ### Uses of IdMultiLabelInstances in main.ml2db.al

    Methods in main.ml2db.al that return IdMultiLabelInstances

    | Modifier and Type | Method and Description |
    | --- | --- |
    |  |  |
    | --- | --- |
    | `IdMultiLabelInstances` | ActiveLearning.`getLabelled()` |
    | `IdMultiLabelInstances` | ActiveLearning.`getLabInstances()` |
    | `IdMultiLabelInstances` | ActiveLearning.`getUnlabelled()` |

    Methods in main.ml2db.al with parameters of type IdMultiLabelInstances

    | Modifier and Type | Method and Description |
    | --- | --- |
    |  |  |
    | --- | --- |
    | `void` | ActiveLearning.`setLabInstances(IdMultiLabelInstances labInstances)` |
    | `void` | ActiveLearning.`setUnlabInstances(IdMultiLabelInstances unlabInstances)` |
  - ### Uses of IdMultiLabelInstances in main.ml2db.al.learning

    Methods in main.ml2db.al.learning that return IdMultiLabelInstances

    | Modifier and Type | Method and Description |
    | --- | --- |
    |  |  |
    | --- | --- |
    | `IdMultiLabelInstances` | ExperimentDetails.`getLabelledInstances()` |
    | `IdMultiLabelInstances` | ExperimentDetails.`getLabInstances()` |
    | `IdMultiLabelInstances` | ExperimentDetails.`getUnlabelledInstances()` |
    | `IdMultiLabelInstances` | ExperimentDetails.`getUnlabInstances()` |

    Methods in main.ml2db.al.learning with parameters of type IdMultiLabelInstances

    | Modifier and Type | Method and Description |
    | --- | --- |
    |  |  |
    | --- | --- |
    | `void` | ExperimentDetails.`setLabInstances(IdMultiLabelInstances labInstances)` |
    | `void` | ExperimentDetails.`setUnlabInstances(IdMultiLabelInstances unlabInstances)` |

    Constructors in main.ml2db.al.learning with parameters of type IdMultiLabelInstances

    Constructor and Description

    `ExperimentDetails(IdMultiLabelInstances unlabelled,
    MulanLearner algorithm,
    java.lang.String distanceFunction,
    DbManagers dbManagers,
    UtilityMetric utilityMetric,
    java.lang.String imagesPath)`

    With set of unlabelled instances (no arff record identifier)
  - ### Uses of IdMultiLabelInstances in main.ml2db.al.utility

    Methods in main.ml2db.al.utility that return IdMultiLabelInstances

    | Modifier and Type | Method and Description |
    | --- | --- |
    |  |  |
    | --- | --- |
    | `IdMultiLabelInstances` | UtilityMetric.`getUnlabelled()` |

    Methods in main.ml2db.al.utility with parameters of type IdMultiLabelInstances

    | Modifier and Type | Method and Description |
    | --- | --- |
    |  |  |
    | --- | --- |
    | `abstract void` | UtilityMetric.`refresh(IdMultiLabelInstances labelled, IdMultiLabelInstances unlabelled, IdInstance queryInstance)` Refresh the set of labelled and unlabelled instances |
    | `void` | UtilityMetric.`setUnlabelled(IdMultiLabelInstances unlabelledInstances)` |
  - ### Uses of IdMultiLabelInstances in main.ml2db.al.utility.confidence

    Methods in main.ml2db.al.utility.confidence with parameters of type IdMultiLabelInstances

    | Modifier and Type | Method and Description |
    | --- | --- |
    |  |  |
    | --- | --- |
    | `void` | ConfidenceMetric.`refresh(IdMultiLabelInstances labelled, IdMultiLabelInstances unlabelled, IdInstance queryInstance)` |
  - ### Uses of IdMultiLabelInstances in main.ml2db.al.utility.globaldistance

    Methods in main.ml2db.al.utility.globaldistance that return IdMultiLabelInstances

    | Modifier and Type | Method and Description |
    | --- | --- |
    |  |  |
    | --- | --- |
    | `IdMultiLabelInstances` | DistancesCalculator.`getInstances()` |
    | `IdMultiLabelInstances` | GlobalDistanceMetric.`getLabelled()` |

    Methods in main.ml2db.al.utility.globaldistance with parameters of type IdMultiLabelInstances

    | Modifier and Type | Method and Description |
    | --- | --- |
    |  |  |
    | --- | --- |
    | `void` | GlobalDistanceMetric.`initialise(IdMultiLabelInstances labelled, IdMultiLabelInstances unlabelled)` |
    | `void` | GlobalDistanceMetric.`refresh(IdMultiLabelInstances labelled, IdMultiLabelInstances unlabelled, IdInstance instanceLabelled)` |
    | `void` | GlobalDistanceMetric.`setInstances(IdMultiLabelInstances labelled, IdMultiLabelInstances unlabelled)` |

    Constructors in main.ml2db.al.utility.globaldistance with parameters of type IdMultiLabelInstances

    Constructor and Description

    `DistancesCalculator(IdMultiLabelInstances instances,
    java.lang.String storageFileName)`

    The distances are not saved to file.

    `DistancesCalculator(IdMultiLabelInstances instances,
    java.lang.String storageFileName,
    boolean writeToFile)`

    `DistancesCalculator(IdMultiLabelInstances instances,
    java.lang.String storageFileName,
    boolean writeToFile,
    weka.core.NormalizableDistance distanceFunction)`
  - ### Uses of IdMultiLabelInstances in main.ml2db.al.utility.hybrid

    Methods in main.ml2db.al.utility.hybrid with parameters of type IdMultiLabelInstances

    | Modifier and Type | Method and Description |
    | --- | --- |
    |  |  |
    | --- | --- |
    | `void` | GlobalDistanceWithConfidenceMetric.`refresh(IdMultiLabelInstances labelled, IdMultiLabelInstances unlabelled, IdInstance instanceLabelled)` |
  - ### Uses of IdMultiLabelInstances in main.ml2db.al.utility.neighbours

    Methods in main.ml2db.al.utility.neighbours that return IdMultiLabelInstances

    | Modifier and Type | Method and Description |
    | --- | --- |
    |  |  |
    | --- | --- |
    | `IdMultiLabelInstances` | NeighbourhoodNetwork.`getUnlabelledInstances()` |

    Methods in main.ml2db.al.utility.neighbours with parameters of type IdMultiLabelInstances

    | Modifier and Type | Method and Description |
    | --- | --- |
    |  |  |
    | --- | --- |
    | `java.util.Vector<weka.core.Instance>` | LinearNNSearchSkipInstanceItself.`getNeighbours(weka.core.Instance instance, IdMultiLabelInstances instanceItselfAndOthers, int kNN, boolean skipItself)` |
    | `void` | LinearMultiLabelNNSearch.`rebuild(IdMultiLabelInstances instances)` |
    | `void` | NeighbourhoodNetwork.`refresh(IdMultiLabelInstances labelled, IdMultiLabelInstances unlabelled, IdInstance instanceLabelled)` |

    Constructors in main.ml2db.al.utility.neighbours with parameters of type IdMultiLabelInstances

    Constructor and Description

    `LinearMultiLabelNNSearch(IdMultiLabelInstances instances)`

    `LinearNNSearchSkipInstanceItself(IdMultiLabelInstances instances)`

    `NeighbourhoodNetwork(IdMultiLabelInstances unlabelledInstances)`

    a list of needed instances
  - ### Uses of IdMultiLabelInstances in main.ml2db.al.utility.pickers

    Methods in main.ml2db.al.utility.pickers with parameters of type IdMultiLabelInstances

    | Modifier and Type | Method and Description |
    | --- | --- |
    |  |  |
    | --- | --- |
    | `IdInstance` | RandomPicker.`getQuery(IdMultiLabelInstances unlabelled)` |
    | `void` | RandomPicker.`refresh(IdMultiLabelInstances labelled, IdMultiLabelInstances unlabelled, IdInstance queryInstance)` |
  - ### Uses of IdMultiLabelInstances in main.ml2db.al.utility.pickers.byfrequency

    Methods in main.ml2db.al.utility.pickers.byfrequency with parameters of type IdMultiLabelInstances

    | Modifier and Type | Method and Description |
    | --- | --- |
    |  |  |
    | --- | --- |
    | `void` | NearestNeighboursByFreqUtility.`initialise(IdMultiLabelInstances unlabelled)` |
    | `void` | NearestNeighboursByFreqUtility.`refresh(IdMultiLabelInstances labelled, IdMultiLabelInstances unlabelled, IdInstance queryInstance)` |
  - ### Uses of IdMultiLabelInstances in main.ml2db.graphviz

    Methods in main.ml2db.graphviz with parameters of type IdMultiLabelInstances

    | Modifier and Type | Method and Description |
    | --- | --- |
    |  |  |
    | --- | --- |
    | `void` | GraphVizArff.`addInstances(IdMultiLabelInstances instances)` |
    | `void` | GraphVizArffColoured.`colourLabelled(IdMultiLabelInstances labelledInstances)` Assigns a colour depending on an integer (from 0 to >4) |
    | `void` | GraphVizArff.`fillDot(IdMultiLabelInstances instances)` |
  - ### Uses of IdMultiLabelInstances in main.ml2db.mulan.learn.crosseval

    Methods in main.ml2db.mulan.learn.crosseval that return IdMultiLabelInstances

    | Modifier and Type | Method and Description |
    | --- | --- |
    |  |  |
    | --- | --- |
    | `IdMultiLabelInstances` | Folds.`getOriginalSet()` |
    | `IdMultiLabelInstances` | CrossInputManager.`getTrainDataSet()` |

    Methods in main.ml2db.mulan.learn.crosseval with parameters of type IdMultiLabelInstances

    | Modifier and Type | Method and Description |
    | --- | --- |
    |  |  |
    | --- | --- |
    | `java.util.Vector<java.lang.Object[]>` | CrossEvaluator.`evaluate(int numFolds, IdMultiLabelInstances trainDataSet, mulan.classifier.MultiLabelLearner learner, boolean savePredictions, boolean saveTrueNegatives, boolean stratify)` Evaluates (runs a cross validation) |
    | `java.util.Vector<java.lang.Object[]>` | CrossEvaluator.`internallyCrossValidate(int numFolds, int seed, IdMultiLabelInstances trainDataSet, mulan.classifier.MultiLabelLearner learner, boolean savePredictions, boolean saveTrueNegatives, boolean stratify)` Evaluates a `MultiLabelLearner` via cross-validation on given data set with defined number of folds. |
    | `void` | CrossExperimenter.`setInputManager(IdMultiLabelInstances trainSet)` |
    | `void` | CrossInputManager.`setTrainDataSet(IdMultiLabelInstances trainDataSet)` |

    Constructors in main.ml2db.mulan.learn.crosseval with parameters of type IdMultiLabelInstances

    Constructor and Description

    `CrossExperimenter(IdMultiLabelInstances trainSet,
    MulanLearner learner,
    DbManagers dbMan,
    java.lang.String resultsFolder)`

    `CrossInputManager(IdMultiLabelInstances trainSet,
    CrossExperimenter experiment)`

    Gets the instances as object.

    `Folds(IdMultiLabelInstances instances,
    int numFolds)`

    `StratifiedFolds(IdMultiLabelInstances instances,
    int numFolds)`
  - ### Uses of IdMultiLabelInstances in main.ml2db.mulan.learn.traintest

    Methods in main.ml2db.mulan.learn.traintest that return IdMultiLabelInstances

    | Modifier and Type | Method and Description |
    | --- | --- |
    |  |  |
    | --- | --- |
    | `IdMultiLabelInstances` | TrainTestInputManager.`getTestDataSet()` |

    Methods in main.ml2db.mulan.learn.traintest with parameters of type IdMultiLabelInstances

    | Modifier and Type | Method and Description |
    | --- | --- |
    |  |  |
    | --- | --- |
    | `void` | TrainTestExperimenter.`setInputManager(IdMultiLabelInstances trainSet)` |
    | `void` | TrainTestInputManager.`setTestDataSet(IdMultiLabelInstances testSet)` |
    | `java.util.Vector<java.lang.Object[]>` | TrainTestEvaluator.`trainTestValidate(IdMultiLabelInstances trainSet, IdMultiLabelInstances testSet, boolean savePredictions, boolean saveTrueNegatives)` |

    Constructors in main.ml2db.mulan.learn.traintest with parameters of type IdMultiLabelInstances

    Constructor and Description

    `TrainTestExperimenter(IdMultiLabelInstances trainSet,
    IdMultiLabelInstances testSet,
    DbManagers dbManagers,
    MulanLearner learner,
    java.lang.String resultsDirectory)`

    `TrainTestInputManager(IdMultiLabelInstances trainSet,
    IdMultiLabelInstances testSet,
    TrainTestExperimenter experimenter)`

    Gets the instances as object.

    `TrainTestInputManager(IdMultiLabelInstances trainSet,
    TrainTestExperimenter experimenter)`

    Gets the instances as object.
  - ### Uses of IdMultiLabelInstances in main.ml2db.mulan.mulanarff

    Methods in main.ml2db.mulan.mulanarff that return IdMultiLabelInstances

    | Modifier and Type | Method and Description |
    | --- | --- |
    |  |  |
    | --- | --- |
    | `IdMultiLabelInstances` | NeighboursPrinter.`getInstances()` |

    Methods in main.ml2db.mulan.mulanarff with parameters of type IdMultiLabelInstances

    | Modifier and Type | Method and Description |
    | --- | --- |
    |  |  |
    | --- | --- |
    | `void` | IdMultiLabelInstances.`addAll(IdMultiLabelInstances instances)` Instances must come from already compatible datasets! |

    Constructors in main.ml2db.mulan.mulanarff with parameters of type IdMultiLabelInstances

    Constructor and Description

    `NeighboursPrinter(IdMultiLabelInstances unlabelledInstances,
    java.util.Set<java.lang.String> desired)`
  - ### Uses of IdMultiLabelInstances in main.ml2db.mulan.mulanarff.database

    Methods in main.ml2db.mulan.mulanarff.database that return IdMultiLabelInstances

    | Modifier and Type | Method and Description |
    | --- | --- |
    |  |  |
    | --- | --- |
    | `static IdMultiLabelInstances` | MulanArffInstancesLoader.`generateIdInstances(int arffRecordId, DbManagers dbMan)` |
  - ### Uses of IdMultiLabelInstances in main.ml2db.mulan.predict

    Methods in main.ml2db.mulan.predict that return IdMultiLabelInstances

    | Modifier and Type | Method and Description |
    | --- | --- |
    |  |  |
    | --- | --- |
    | `IdMultiLabelInstances` | PredictionsSaver.`getBaseDataToBePredicted()` |
    | `IdMultiLabelInstances` | PredictionsSaver.`getFullTrainingSet()` |

    Methods in main.ml2db.mulan.predict with parameters of type IdMultiLabelInstances

    | Modifier and Type | Method and Description |
    | --- | --- |
    |  |  |
    | --- | --- |
    | `int[]` | PredictionsSaver.`generatePredictions(mulan.classifier.MultiLabelOutput multiLabelOutput, IdInstance instance, IdMultiLabelInstances baseData, boolean[] trueLabels)` |

    Constructors in main.ml2db.mulan.predict with parameters of type IdMultiLabelInstances

    Constructor and Description

    `PredictionsSaver(uk.ac.standrews.utils.main.database.DbManager resultsDbManager,
    int trainArffId,
    int testArffId,
    IdMultiLabelInstances fullTrainSet,
    IdMultiLabelInstances fullTestSet,
    mulan.data.MultiLabelInstances trainSet,
    mulan.data.MultiLabelInstances testSet,
    MulanLearner baseLearner,
    mulan.classifier.MultiLabelLearner trainedLearner,
    boolean saveTrueNegatives,
    int fold)`
  - ### Uses of IdMultiLabelInstances in main.ml2db.weka

    Methods in main.ml2db.weka that return IdMultiLabelInstances

    | Modifier and Type | Method and Description |
    | --- | --- |
    |  |  |
    | --- | --- |
    | `IdMultiLabelInstances` | JaccardDistanceStored.`getIdInstances()` |

    Constructors in main.ml2db.weka with parameters of type IdMultiLabelInstances

    Constructor and Description

    `JaccardDistanceStored(IdMultiLabelInstances idInstances)`
  - ### Uses of IdMultiLabelInstances in test.ml2db.al.utility.confidence

    Methods in test.ml2db.al.utility.confidence that return IdMultiLabelInstances

    | Modifier and Type | Method and Description |
    | --- | --- |
    |  |  |
    | --- | --- |
    | `static IdMultiLabelInstances` | ConfidenceCalculatorTest.`getInstancesOfTrainedLerner1()` |
    | `static IdMultiLabelInstances` | ConfidenceCalculatorTest.`getInstancesOfTrainedLerner2()` |

    Methods in test.ml2db.al.utility.confidence with parameters of type IdMultiLabelInstances

    | Modifier and Type | Method and Description |
    | --- | --- |
    |  |  |
    | --- | --- |
    | `static mulan.classifier.MultiLabelLearner` | ConfidenceCalculatorTest.`getTrainedLearner(IdMultiLabelInstances instances)` |
  - ### Uses of IdMultiLabelInstances in test.ml2db.mulan.mulanarff

    Methods in test.ml2db.mulan.mulanarff that return IdMultiLabelInstances

    | Modifier and Type | Method and Description |
    | --- | --- |
    |  |  |
    | --- | --- |
    | `static IdMultiLabelInstances` | IdMultiLabelInstancesTest.`getIdMultiLabelInstancesTestset()` |
    | `static IdMultiLabelInstances` | IdMultiLabelInstancesTest.`getIdMultiLabelInstancesTrainset()` |

    Methods in test.ml2db.mulan.mulanarff with parameters of type IdMultiLabelInstances

    | Modifier and Type | Method and Description |
    | --- | --- |
    |  |  |
    | --- | --- |
    | `static Statistics` | StatisticsTest.`getStats(IdMultiLabelInstances data)` |

- Overview
- Package
- Class
- Use
- Tree
- Deprecated
- Index
- Help

- Prev
- Next

- Frames
- No Frames

- All Classes
